# Identification, Isolation and Genomic Characterization of an Emerging Shamonda Virus Variant Associated with Bovine Disease in Switzerland

**DOI:** 10.3390/v18101086

**Published:** 2026-10-01

**Authors:** Michele Wyler, Karin Darpel, Rémy Berset, Sandra Renzullo, Helen Huber, Jessica Bauer, Judith Peter-Egli, Claudia Bachofen, Jakub Kubacki

**Affiliations:** 1 Institute of Virology and Immunology, 3147 Mittelhaeusern, Switzerland; 2 Department of Infectious Diseases and Pathobiology, Vetsuisse Faculty, University of Bern, 3012 Bern, Switzerland; 3 Cattle Health Service Switzerland (Rindergesundheit Schweiz RGS), 3052 Zollikofen, Switzerland

**Keywords:** *Orthobunyavirus*, emerging virus, virus pathogen, Shamonda virus

## Abstract

In July 2026, an outbreak of a cattle illness characterized by high fever, diarrhea and reduced milk production, often affecting whole herds, was detected across several regions of northern Switzerland. To investigate the cause of the outbreak, a broad range of diagnostic methods was applied, including pathogen-specific RT-qPCRs, ELISA, serum neutralization test, virus isolation and metagenomic next-generation sequencing. A Shamonda virus (SHAV) was detected in samples from affected cattle, followed by successful virus isolation and complete genome sequencing of all three segments. Viral RNA was also detected in tissue samples from aborted calves, including the brain and spleen, supporting the need for further investigation of the virus in the context of reproductive disease. These findings document the first detection and molecular characterization of a SHAV in Switzerland and demonstrate its widespread circulation in cattle during the 2026 outbreak.

## 1. Introduction

Viruses of the genus *Orthobunyavirus* are arthropod-borne viruses within the family *Peribunyaviridae*, characterized by a segmented, negative-sense RNA genome [1]. The genome has a large (L), a medium (M) and a small (S) segment, encoding the RNA-dependent RNA polymerase, the glycoproteins, and the nucleocapsid protein, respectively. The segmented genome allows genetic reassortment, when two *Orthobunyaviruses* co-infect the same host or vector, potentially contributing to viral evolution and emergence. The major antigenic group within the genus is the unofficial Simbu serogroup that comprises clade B viruses of considerable veterinary relevance [2,3,4]. These include, among others, the Akabane virus (AKAV), the Schmallenberg virus (SBV), the Aino Virus (AINOV), the Shuni Virus (SHUV) and the Shamonda virus (SHAV). Several members of this group have been associated with reproductive diseases, congenital abnormalities, abortions and stillbirths in cattle, small ruminants and, in the case of SHUV, neurological disease in horses. All are primarily transmitted by *Culicoides* biting midges, and their epidemiology depends on vector abundance, seasonal activity and geographic distribution, factors that can facilitate the introduction and establishment of new *Orthobunyaviruses* in susceptible ruminant populations.

SBV represents a particularly important example of the ability of *Orthobunyaviruses* to emerge in previously unaffected geographical regions. The virus was detected for the first time in Germany in 2011 in plasma samples from cattle with fever and reduced milk production [5]. This provided a notable case for the discovery of a novel member of the Simbu serogroup virus via metagenomic sequencing. Following initial detection, SBV spread rapidly across Europe, causing mild or transient clinical symptoms in adult cattle, whereas infection of fetuses during the critical stage of gestation resulted in congenital abnormalities including arthrogryposis, hydranencephaly, abortion and stillbirths. SBV has been proposed to have a reassortant origin, with its S and L segments closely related to SHAV, whereas its more divergent M segment has been proposed to have a distinct SATV-like origin [6]. As the M segment encodes the viral glycoproteins Gn and Gc, sequence divergence in this segment may result in antigenic differences between SBV and SHAV. SHAV was originally detected and isolated from cattle and biting midges in Nigeria in the 1960s, subsequently detected after 40 years in biting midges and blood samples from cattle in Japan in 2002, and serological evidence of a Shamonda-like virus has been reported from New South Wales, Australia in 2019 [7,8]. Despite this close evolutionary relationship, the epidemiology and geographic distribution of SHAV and Shamonda-like viruses remain unknown.

In the second week of July 2026, the Cattle Health Service Switzerland (Rindergesundheit Schweiz RGS) recorded a significant rise in reports of fever and diarrhea accompanied by a simultaneous decline in milk yield in cattle, predominantly in dairy cows. The first reports came from the western part of Switzerland and were followed by reports across central Switzerland as far as the eastern canton of Thurgau. Due to the rapid and apparently epidemic spread of clinical symptoms, uncertainty grew amongst both veterinarians and farmers. Similar clinical signs, initially thought to be related to the intensive heatwave, were observed in southern Germany and eastern France. Initial virological diagnostic testing did not identify any known viral pathogen as cause of the observed disease. SBV was initially considered; however, samples from a few animals submitted for SBV diagnostics all tested negative by SBV RT-qPCR targeting the S segment. Taking into consideration the short viraemic period of SBV infections, targeted serological investigations alongside sampling of recent acutely ill animals were undertaken on three selected farms with notable disease rates in adult dairy cows. Indeed, high seroprevalence of SBV antibodies was detected on all three farms from geographically distinct regions. Further investigation of antibody-negative animals or low antibody-positive acute clinical animals in these herds using a broad range pan-Simbu RT-qPCR revealed that several animals were positive for the viral genome. The subsequent metagenomic sequencing of four samples from two affected farms identified an *Orthobunyavirus*. Complete genome sequences of all three segments were obtained from all four samples and demonstrated that the virus is most closely related to SHAV but is generally distinct from previously characterized sequences. The Swiss sequences showed high genomic similarity with the viruses concurrently sequenced in Germany and France, indicating the circulation of a previously uncharacterized Shamonda-like *Orthobunyavirus* in Western Europe [9,10].

Following the emergence of SBV about 15 years ago, the identification of another Simbu serogroup *Orthobunyavirus* associated with disease in cattle in several European countries raises concerns of the constant establishment of this virus and, with SBV still circulating, of potential reassortments between them [11].

The present study describes the detection, molecular characterization and phylogenetic classification of a novel Shamonda-like virus and provides the first evidence of its circulation in Switzerland. Virus isolation and whole-genome sequencing were utilized to characterize the virus at the genomic level and to establish its phylogenetic relatedness to SHAV, SBV and other veterinary-relevant members of the genus *Orthobunyaviruses* based on all three segments and assess its potential geographic distribution and possible impact on animal health.

## 2. Materials and Methods

### 2.1. Laboratory Timeline

During the week of 20–24 July, an increasing number of inquiries were received concerning dairy cattle presenting with fever, diarrhea, and a decrease in milk production. SBV infection was repeatedly considered based on the clinical presentation. However, initial samples were mainly submitted from single animals with fully developed clinical signs, such as diarrhea, for several days, and all samples tested negative by SBV-specific RT-qPCR [12]. Given that SBV viremia is reported to be relatively short, with viral RNA generally detectable for approximately six days only, it was considered possible that samples had been collected after the viremic phase and that the optimal diagnostic window had therefore been missed. To further elucidate whether SBV needed to be considered as a possible cause of the observed disease, primary veterinary practitioners, in association with the Cattle Health Service Switzerland (Rindergesundheit Schweiz RGS), carried out census sampling of adult cattle on three farms, each with high incidence of disease and representing the regions of western, central and eastern Switzerland.

On Friday, 24 July serum samples from 27 adult dairy cows of a herd in the canton of Jura (western Switzerland) were sent, followed the following week by serum samples of five calves born in 2026. On Tuesday, the 28 July samples from a farm in the canton of Thurgau (eastern Switzerland) taken from 29 dairy cows, including four acutely affected animals plus three young calves, were received. Finally, on Friday, the 31st of July samples from 38 dairy cows, including three acutely ill animals, as well as samples from 10 calves born in 2026, were sent from a farm in the canton of Lucerne (central Switzerland). All samples were analyzed for SBV-specific antibodies as described below.

### 2.2. Serological Analysis

Serum samples of the animals from the three herds were analyzed with a commercially available indirect ELISA (ID Screen^®^ Schmallenberg Virus Multi-species, IDvet, Grabels, France), according to the manufacturer’s instructions. To further investigate the specificity of the antibody response, a SBV serum neutralization test (SNT) established for routine diagnostic at IVI was performed on a subset of 10 serum samples representing different ELISA reactivity levels. However, due to a lack of other *Orthobunyaviruses*, serum samples were only tested for their ability to neutralize SBV (Isolate BH80/11-4 originally isolated from cattle serum and received from H. Schirrmeier, Friedrich-Loeffler Institute). Briefly, neutralization assay was carried on serial two-fold dilution of sera from 1:4 until 1:256 in four wells with a total volume of 50 μL per well. Then, 50 μL virus diluted in medium to contain approximately 200 TCID50 (confirmed by back-titration) was added to each well containing serum and incubated for 24 h. Following incubation, the serum–virus mixtures were transferred to BHK-21 cells plates and further incubated for 72 h in 37 °C with 5% CO_2_; presence/absence of CPE was recorded and neutralization titer was calculated using the Spearman–Karber formula. Neutralizing titer was reported as the highest dilution at which neutralization was observed.

### 2.3. RT-qPCRs

Nucleic acid from selected serum/EDTA-Blood samples was extracted using NucleoMag^®^ VET Kit (Macherey Nagel, Düren, Germany) on a KingFisher automated extraction system (Thermo Fisher Scientific, Waltham, MA, USA), according to the manufacturer’s instructions. Following RNA extraction, all selected samples were tested for SBV virus RNA using published SBV-specific RT-qPCR targeting the S segment. However, SBV-S RNA was not detected in any of the tested animals, including the clinically affected animals and those with negative or doubtful SBV antibody results. Therefore, the samples were further analyzed using a pan-Simbu RT-qPCR (targeting S segment), and SBV-specific RT-qPCRs targeting the conserved L and the M segments according to published assays [12,13]. Furthermore, four pan-Simbu RT-qPCR positive samples were selected for whole-genome sequencing (WGS), while seven samples were taken forward for virus isolation to further characterize the detected virus.

### 2.4. Virus Isolation

Six sera positive in pan-Simbu RT-qPCR and one EDTA-Blood with CT-values between 20.9 and 27.8 were used in an attempt to isolate the virus using SK-6 and BHK-21 cells. Cells were cultured in 25 cm^2^ cell culture flasks. Both BHK-21 and SK-6 were inoculated with 2 mL of the diluted 1:4 sera/blood in GMEM or MEM medium plus Penicillin/Streptomycin antibiotics (all Gibco, LuBio Science GmbH, Luzern, Switzerland) for BHK-21 and SK6 respectively.

The cells were incubated for 2 h at 37 °C. To cell cultures inoculated with serum samples, 8 mL of the respective media, plus antibiotics and 2% fetal bovine serum in BHK-21 or 2% horse serum in SK6 cells (Gibco, LuBio Science GmbH, Luzern, Switzerland), was added with the inoculum remaining. The EDTA inoculum was carefully removed, the cells were washed once, and 10 mL volumes of the respective media were added as described above. All flasks were incubated at 37 °C with 5% CO_2_. The cells were microscopically controlled daily for CPE.

Due to timing constraints, flasks were already frozen at −70 °C after 48 h and freeze-thawed twice. Supernatants were clarified by low-speed centrifugation, stored in aliquots at −70 °C, and used for an additional passage P2. Inoculation of BHK-21 and SK6 was carried out using 2 mL of P1-supernatant directly as the inoculum, incubated for 72 h at 37 °C with 5% CO_2_, and harvested as described above.

Three P2 cell cultures which had shown the most promising CPE on BHK cells were carried forward for virus titration, and titers expressed as TCID50/mL in BHK-21 cells were determined for virus stocks. Furthermore, the expected increase in viral RNA upon successful virus amplification was analyzed in the P1 cell culture passage by testing harvested supernatants using the pan-Simbu RT-qPCR assay.

### 2.5. Next-Generation Sequencing

First, the EDTA-Blood sample (CT 21.2) was gently mixed by inverting the tube. A volume of 1.5 mL was transferred to a new tube and centrifuged for 10 min at 1600× *g*. The buffy coat was then carefully pipetted off together with a small volume of plasma. Three serum samples (CT 20.9–22.9) and one EDTA-Blood sample were relatively enriched for virus particles as previously described [14]. The EDTA-Blood sample 261216_36 and serum sample 261216-18 were taken from the same animal. Subsequently, the samples were extracted using TRIzol™ Reagent (Thermo Fisher Scientific) at a sample-to-reagent ratio of 1:3, according to the manufacturer’s instructions, and β-mercaptoethanol (1%) was added to the TRIzol™ reagent to inactivate nucleases used during the previous step. Reverse transcription, second strand synthesis, and random amplification were performed as previously described using the SISPA (sequence-independent single-primer-amplification) method [14]. The DNA was divided into two aliquots; one aliquot was sheared by sonication (E220 Ultrasonicator, Covaris, MA, USA) at the NGS Platform in Bern, targeting fragments of 500 bp length, and the other aliquot was used directly for library preparation. Sequencing libraries were made using the NEBNext Ultra II DNA Library Prep Kit for Illumina (New England Biolabs, Ipswich, MA, USA) and barcoded using NEBNext Multiplex Oligos (96 Unique Dual Index Primer Pairs; New England Biolabs, Ipswich, MA, USA). Finally, libraries were sequenced using the NovaSeq™ X Series 1.5B Reagent Kit (300 Cyc), on an Illumina NovaSeq X Benchtop sequencer with loading concentration of 160 pM (Illumina, San Diego, CA, USA) at the NGS Platform, Bern, Switzerland.

### 2.6. Data Analysis

To identify the best matching reference and to consider possible reassortments, all available *Orthobunyavirus* sequences of all three segments were downloaded from NCBI GenBank (19 August 2026). To reduce redundancies, the sequences were clustered using cd-hit-est (v.4.8.1) using the options -c 0.99 -n 10 -d 0 -M 500000 [15]. The raw reads were mapped to the resulting clustered reference sequences using bowtie2 (v. 2.5.5) with options -k 20 [16]. The most appropriate reference for each segment was identified using samtools coverage (v.1.23, ref. [17]), resulting in the accession numbers OZ502471.1–OZ502473.1. Consequently, the raw reads were mapped again to the newly identified accession using bowtie2 (v. 2.5.5) with the option –very-sensitive-local [16]. Subsequently, new majority consensus sequences were called with samtools consensus (v.1.23) using options --show-ins no --show-del no --mode simple --call-fract 0 -f fasta [17]. Reads from high-coverage samples were subsampled using samtools view (v.1.23, ref. [17]).

Segment specific alignments were made using mafft (v.7.490) with --op 5 options [18]) using representative sequences from NCBI GenBank. Briefly, the publicly available sequences were clustered using cd-hit-est (v.4.8.1) using the options -c 0.999 -n 10 -d 0 -M 500000 [19].

Pairwise distances were calculate using famsa -dist_export (v.2.4.1, ref. [20]) on alignments trimmed with trimal (v.1.5, ref. [21]) with options -gt 0.9. Genetic distances between samples and publicly available Shamonda and Schmallenberg viruses were tested using a linear mixed model implemented in lme4 (v.1.1.37, ref. [22]). Briefly, a model was fitted with genetic distance as a response variable with the interaction between segment and virus group as a predictor and the individual samples as a random effect. Subsequent pairwise comparisons were Tukey-adjusted using the emmeans package (v.1.11.2, ref. [23]). Visualization of phylogenetic trees was made with the ggtree package (v.3.10.1) using a circular layout and branch length capped to 0.02 to limit compression effects from distant rooting [24]. To correct for abundance differences in publicly available sequences, redundant SBV tree tips were trimmed using Treemer (v0.3) with option -r 1 -RTL 0.9 and -r 1 -RTL 0.975 for segment S and M respectively. Rooting of the tree was made using Simbu serogroup clade B sequences from the ICTV taxonomy (MSL #41, http://ictv.global/taxonomy, accessed on 19 August 2026).

### 2.7. Geographic Distribution of Positive Samples

Geographic distribution was plotted using the packages sf (v.1.1, and ggplot 2 (v.3.5.2) [25,26]. For each farm, the postal code associated with the submitted samples was used as the representative spatial unit.

## 3. Results

### 3.1. Serological Analysis

Serological analysis revealed high levels of anti-SBV antibodies across all three affected farms. On the farm from canton Jura, all 27 adult dairy cows were seropositive, corresponding to a seroprevalence of 100% within the sampled group. No acutely ill adult animal was present anymore at the time of sampling. Additionally, five calves born in 2026 were also tested, of which four were SBV antibody-positive, while one calf remained negative for SBV antibodies.

In Thurgau, 26 out of 29 sampled dairy cows were seropositive, two had indeterminate results, and one was seronegative, corresponding to an overall seroprevalence of approximately 90%. Three calves born in 2026, however, were seronegative, despite two being born to antibody-positive dams. The four acute clinical animals were all SBV antibody-positive.

In Lucerne, 36 out of 38 sampled cows were found to be positive by the SBV ELISA, corresponding to a seroprevalence of approximately 95%. Among the 10 calves, two were seropositive and eight negative. Several antibody-negative calves younger than 6 months were the offspring of antibody-positive dams. Of the three acute clinical animals, one animal was still SBV antibody-negative, while the other two had detectable levels of SBV antibodies.

The total absence of maternal antibodies in some young calves born to SBV antibody-positive mothers suggested that the dams were very recently infected (after calving), and since all farms had a direct dam–calf colostrum regime, this provided the first tentative evidence for recent circulation of SBV, or a related virus.

The SBV ELISA results of 10 sera showed considerable variation in antibody reactivity, with sample-to-positive (S/P) values ranging from 10 to 222%. Corresponding SBV SNT titers ranged from <1:4 to >1:256. (Table 1) All samples but one were classed positive for SBV Antibodies by ELISA, and also had detectable neuralization titers against SBV, albeit with a marked range in SNT titers that was not always aligned to the S/P% values obtained. However, sample 261,239.44, which was found positive for SBV antibodies by ELISA without a detectable SNT titer, had been taken from a calf born in 2026; hence, the fact that maternal antibodies were detected cannot be excluded. Importantly, the serological assays used in this study cannot reliably differentiate between antibodies and closely related *Orthobunyaviruses*, and were therefore interpreted in conjunction with the molecular and virological findings. Given the concurrent detection of a novel Shamonda-like virus in the affected animals, the observed SBV ELISA and SNT reactivity was therefore considered to most likely be from serological cross-reactivity with the newly identified virus, rather than SBV infection.

### 3.2. RT-qPCRs and Geographic Distribution

Overall, the high seroprevalence observed across the three geographically distinct herds, together with high antibody levels, could potentially indicate widespread exposure to SBV. However, this finding was not consistent with the lack of SBV RNA detection—even in clinically affected animals. Therefore, the possibility of the presence of an alternative *Orthobunyavirus* was taken into consideration and prompted further molecular investigation.

The initial molecular investigation of 16 animals, selected from either clinically affected animals or animals with weak/negative results from the SBV ELISA, revealed that six animals were found positive by pan-Simbu RT-qPCR and by the SBV L-segment RT-qPCR, while remaining negative in RT-qPCR assays targeting the SBV M and S segments. Of these six animals, five were clinically affected animals for which EDTA-Blood and sera samples were available. Four out of six animals were already weakly SBV antibody-positive, and overall, ELISA reactivity values were 9%, 40%, 72%, 83%, 88%, and 125% respectively. The discordant PCR pattern, with detection of the conserved L segment but absence of the M and S segments signals, underpinned the suspicion evoked by the serological results of the presence of an SBV-related *Orthobunyavirus* and initiated whole-genome sequencing.

**Figure 1:**
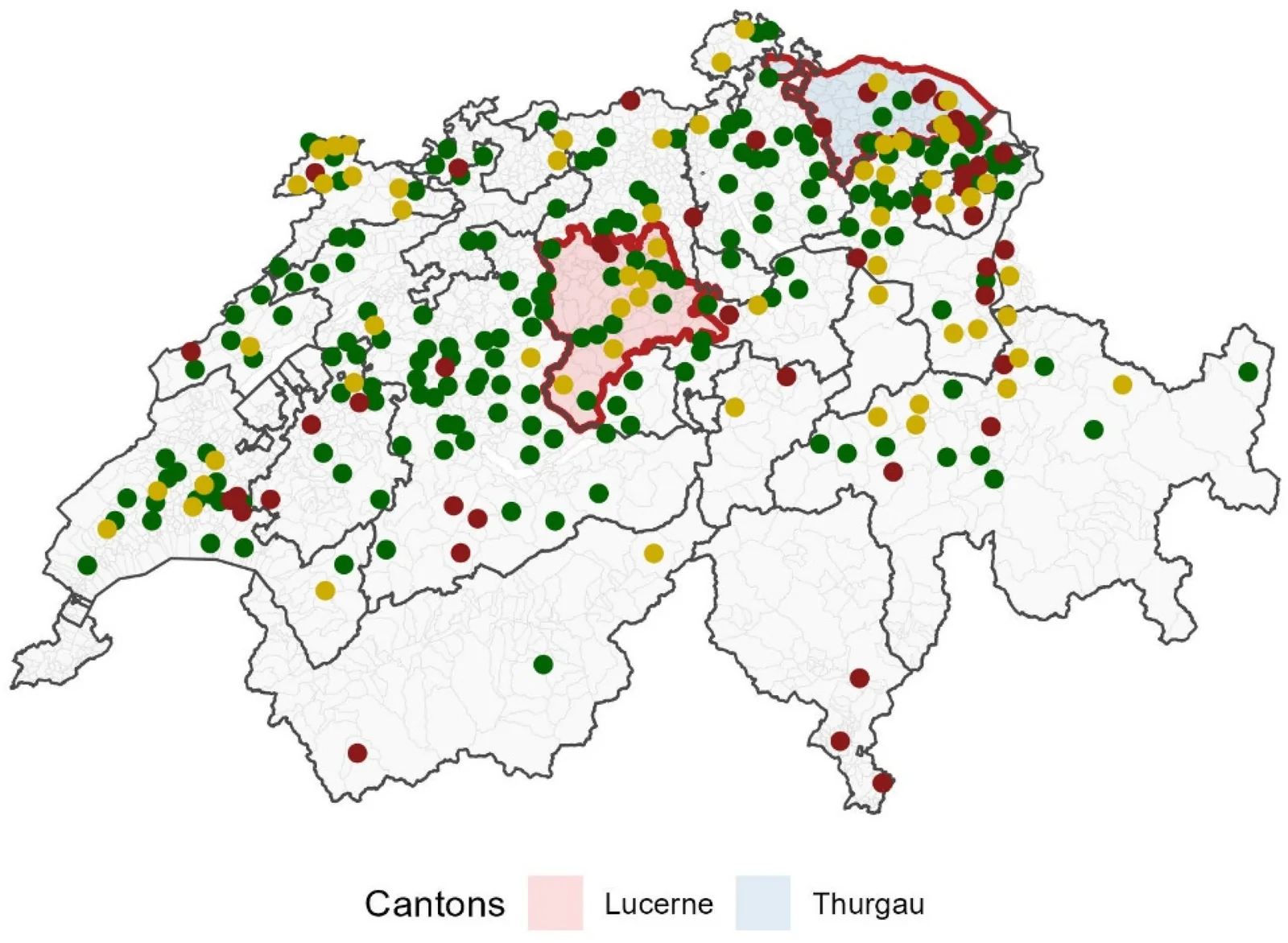
Geographic distribution of analyses performed up to 25 August 2026. Red dots indicate municipalities with SHAV-positive farms, green dots indicate municipalities with SHAV-negative farms, and yellow dots indicate municipalities with both SHAV-positive and SHAV-negative farms. Municipalities are indicated with a gray border. Cantons with full genome sequences are marked with red and blue.

As of 25 August 2026, samples from 280 farms had been submitted for diagnostic testing. A total of 155 animals from 110 herds had been tested as positive for the novel *Orthobunyavirus* by pan-Simbu RT-qPCR, while all samples were tested as negative by specific SBV-S segment RT-qPCR results. Positive herds were identified across 21 Swiss cantons (Figure 1), demonstrating the rapid and geographically widespread emergence of the virus through Switzerland. The spatial distribution of the submitted samples indicated a west-to-east pattern. Interestingly, the submitted samples represent a broad altitudinal range from approximately 200 m above sea level (a.s.l.) in Sant’Antonino (Canton Ticino) to approximately 1250 m a.s.l. in Frutigen (Canton Bern), encompassing regions characterized by sub-Mediterranean to Alpine climatic conditions. Additionally, to date, 22 aborted calves have been tested as positive either in the spleen, brain or both, providing further evidence of association with reproductive disease in cattle.

### 3.3. Virus Isolation

After 48 h of incubation, cytopathic effect was not visible either on BHK-21 or SK-6 cells. However, all harvested cell culture supernatants were confirmed to be positive by pan-Simbu RT-qPCR, with all samples inoculated onto BHK cells showing a clear decrease in CT-values, indicating successful viral replication. In contrast, viral replication in SK-6 cells was less efficient even in passage 2, and these cultures were excluded from further testing and production. Following the second passage, the three samples exhibiting the most pronounced CPE (approximately 80–100%) in BHK cells were chosen for virus titration. Virus titers obtained on BHK cells ranged from 10^5.8^ to 10^6.2^ TCID50/mL.

### 3.4. Next-Generation Sequencing

A total of 880 million raw reads were generated in the sequencing run, ranging from 158 to 325 million reads per sample. Of these, 223 million reads were mapped to the SHAV genome, with the mapped reads ranging from 33,490 reads in the EDTA-Blood sample TG_261216_36 to 146 million reads in the serum sample LU_261239_3. The mean sequencing depth for SHAV was 270,616x, 741,480x, 487,052x, and 358x for samples LU_261239_1, LU_261239_3, TG_261216_18, and TG_261216_36, respectively (Table 2).

**Figure 2:**
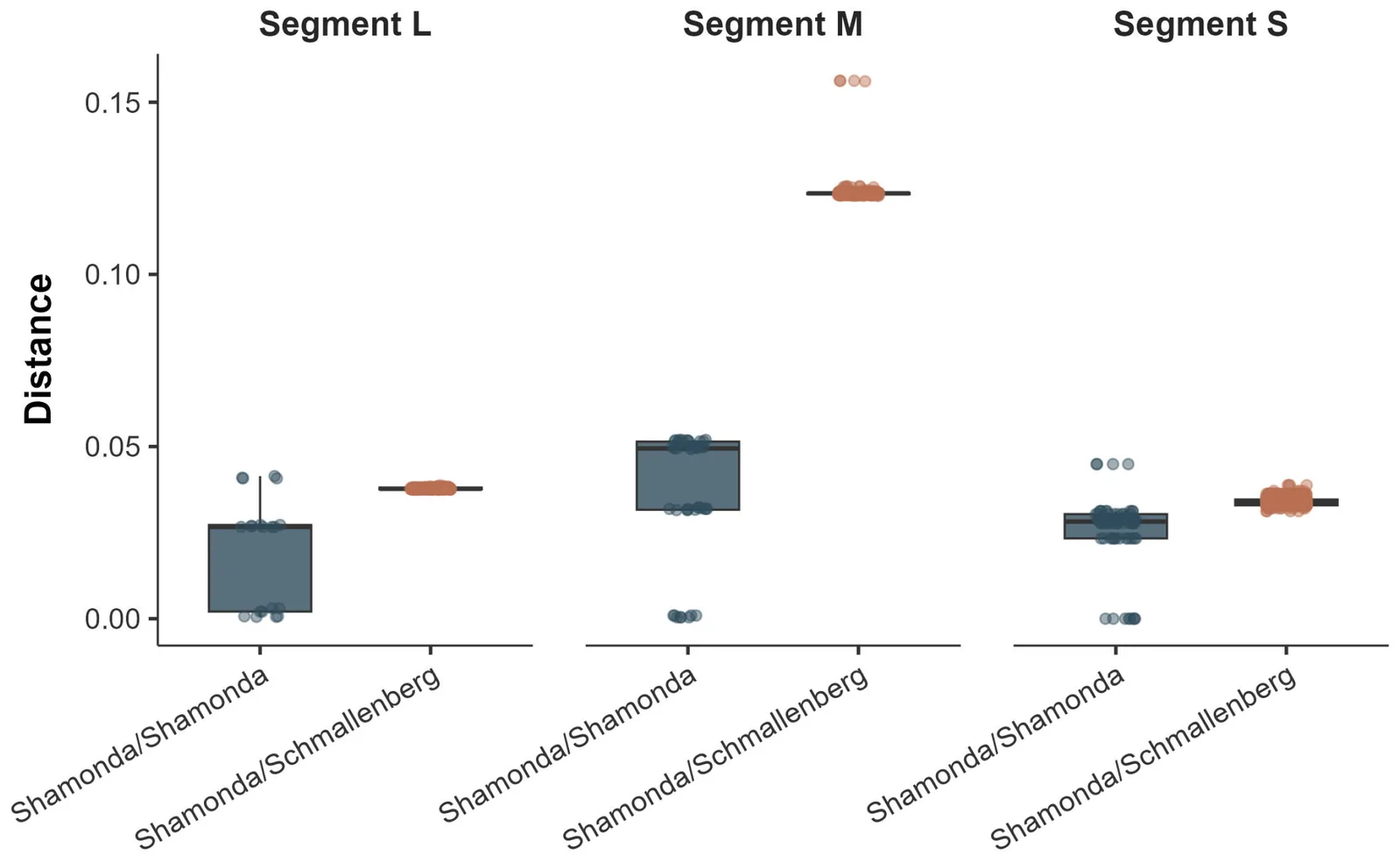
Segment-wise genetic distance of newly sequenced sequences to publicly available known SHAV and SBV sequences available from GenBank. Distances calculated using famsa on trimmed alignments. Boxplots show the median and interquartile range (IQR; 25th–75th percentiles), with whiskers extending to 1.5 × IQR and individual points representing observations.

To statistically assess the group assignment of the newly sequenced samples, we compared their genetic distances to publicly available SHAV and SBV sequences (retrieved on the 15 of August). Distances between virus species were significantly different for all segments (pval < 0.01, Figure 2). However, the magnitude of the distance differed between segments with the M segment displaying the greatest difference (0.0873, pval < 0.01). Furthermore, sequence identity analysis revealed that the novel sequences share only limited identity with the SBV reference strain (55%; NC_043584.1) for the M segment, contrasting sharply with the 91% identity observed against the SHAV reference (NC_018467.1). For the remaining genomic segments, the identity variances between SHAV and SBV were less pronounced, ranging from 89% to 96%. Overall, these results indicate a significantly closer genetic proximity to SHAV than to SBV.

**Figure 3:**
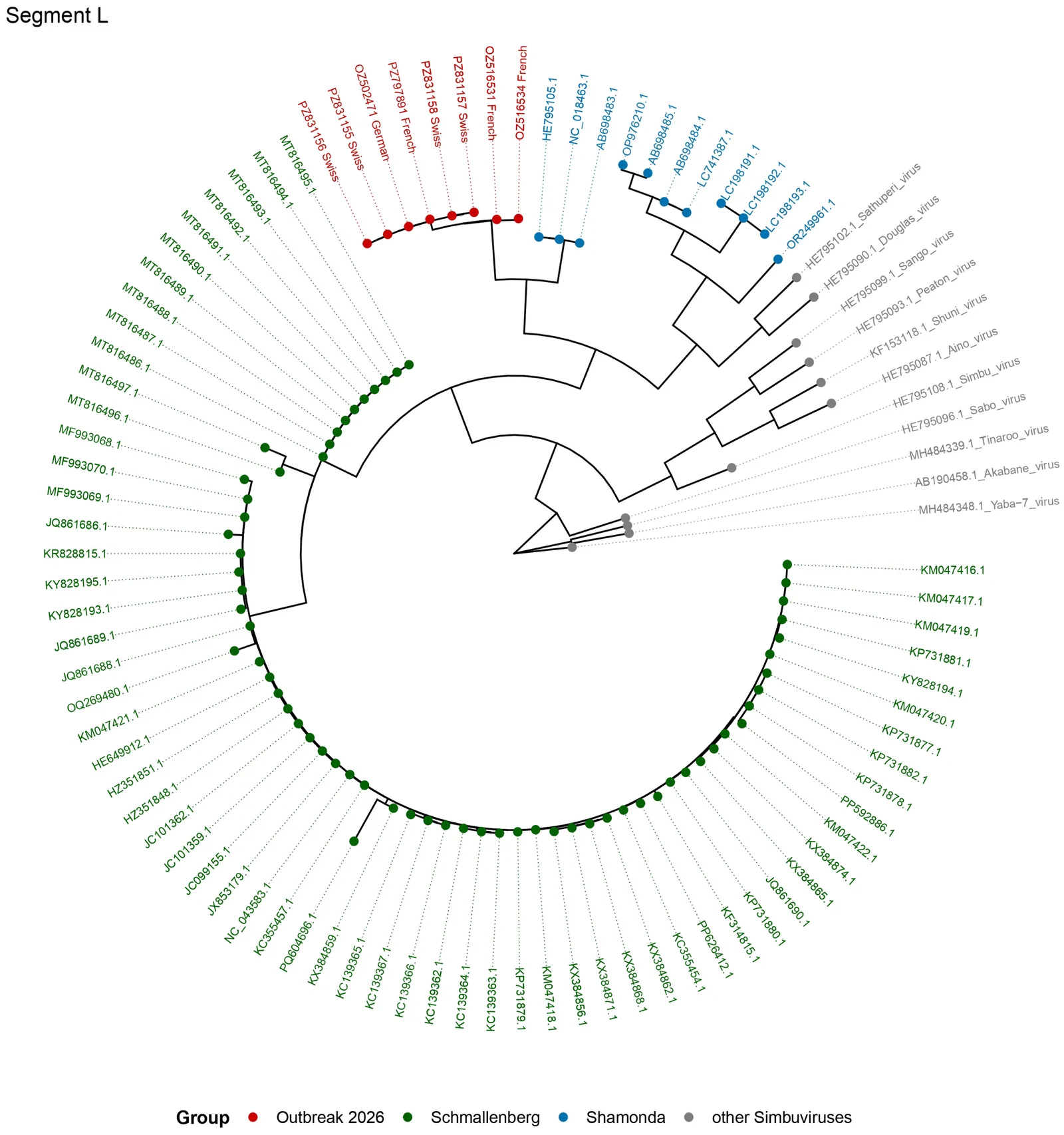
Phylogenetic tree of the L segment. The tree was constructed with the fasttree approximately maximum-likelihood method. Publicly available SBV sequences are indicated in green, whereas publicly available SHAV sequences are shown in blue. The red labels indicate the samples from the 2026 outbreak. Gray labels are representative samples of other *Orthobunyavirus* viruses from the Simbu serogroup from the ICTV taxonomy used for tree rooting. IDs correspond to GenBank entries.

**Figure 4:**
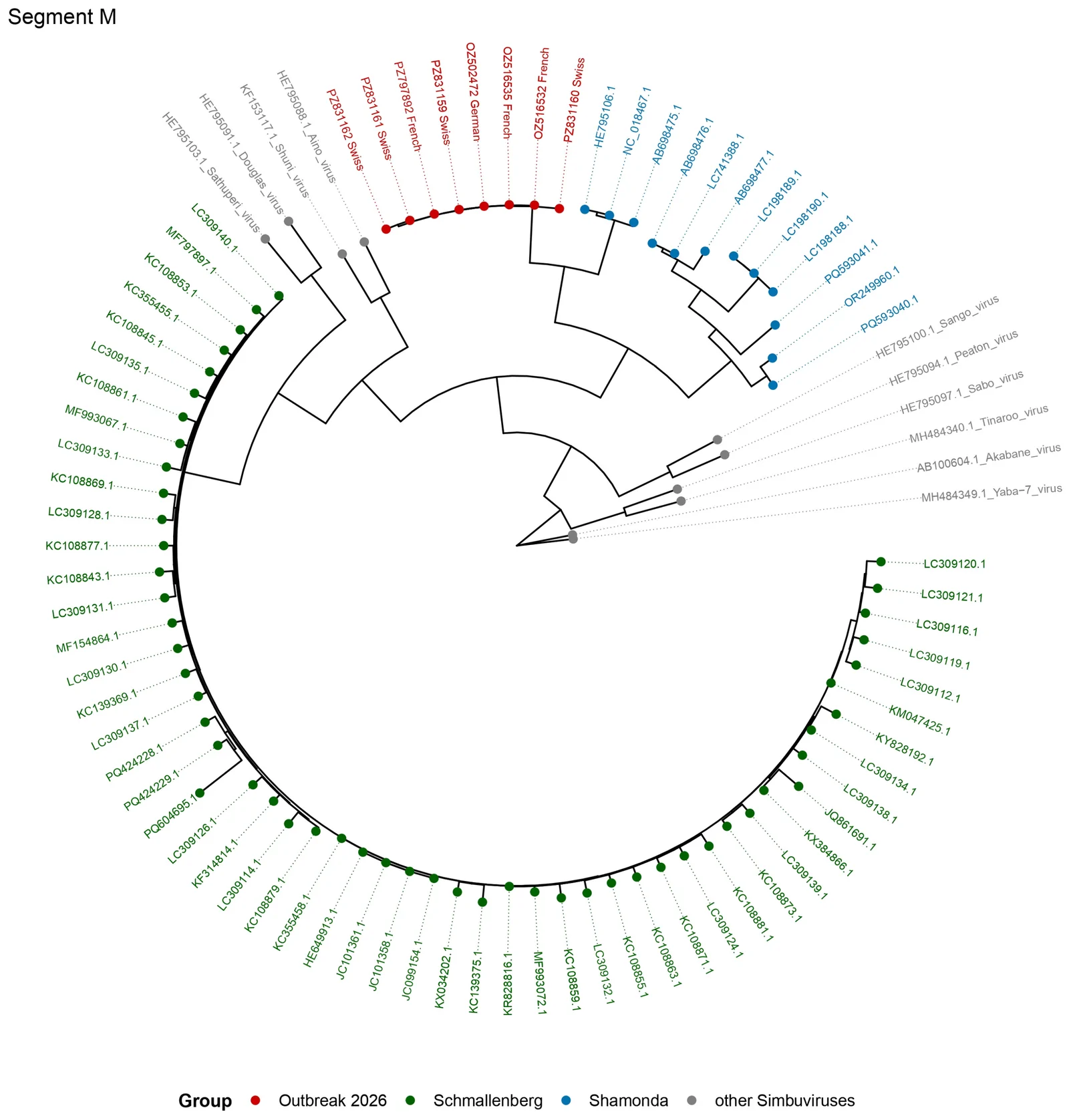
Phylogenetic tree of the M segment. The tree was constructed with the fasttree approximately maximum-likelihood method. Publicly available SBV sequences are indicated in green, whereas publicly available SHAV sequences are shown in blue. The red labels indicate the samples from the 2026 outbreak. Gray labels are representative samples of other *Orthobunyavirus* viruses from the Simbu serogroup from the ICTV taxonomy used for tree rooting. IDs correspond to GenBank entries.

**Figure 5:**
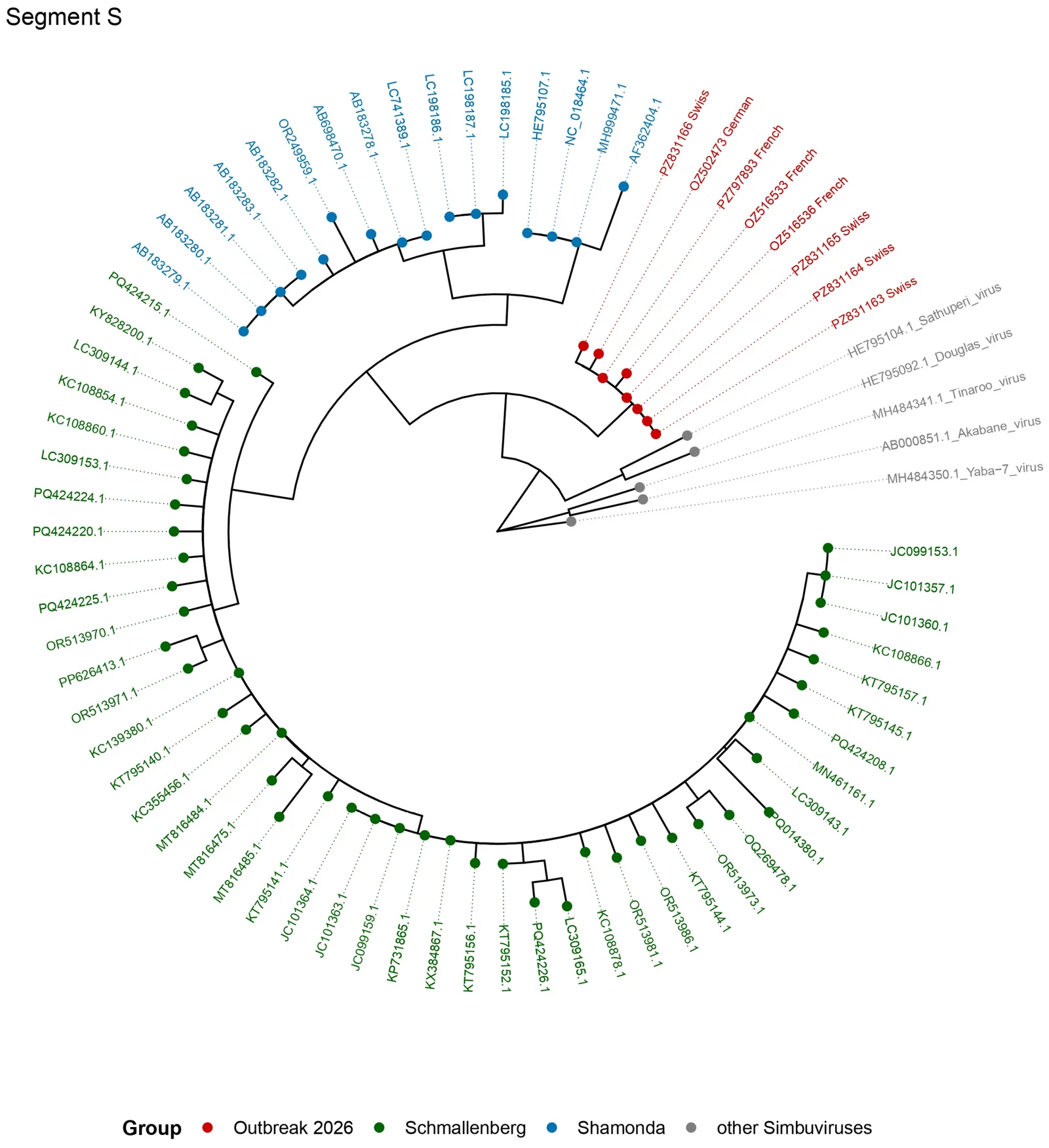
Phylogenetic tree of the S segment. The tree was constructed with the fasttree approximately maximum-likelihood method. Publicly available SBV sequences are indicated in green, whereas publicly available SHAV sequences are shown in blue. The red labels indicate the samples from the 2026 outbreak. Gray labels are representative samples of other *Orthobunyavirus* viruses from the Simbu serogroup from the ICTV taxonomy used for tree rooting. IDs correspond to GenBank entries.

Segment-wise phylogenetic analyses to publicly available sequences revealed a closer relationship between the 2026 outbreak sequences and previously reported SHAV sequences compared to SBV (Figure 3, Figure 4 and Figure 5). However, the extent of this relationship is strongly segment-specific. Notably, the 2026 outbreak sequences of the S segment formed a distinct subclade within the broader Shamonda virus lineage (Figure 4).

A similar pattern is visible at the amino acid level. In fact, the Swiss sequences share 99.57% identity with both the SBV and SHAV reference nucleoproteins. However, when comparing the envelope glycoproteins, the Swiss sequences share 97.65% identity with the SHAV reference protein, but only 35.21% identity with the SBV equivalent.

## 4. Discussion

The serological findings provided the first important indication for a possible widespread outbreak of SBV (or antigenic-related viruses) in Switzerland. Across three geographically distinct farms, SBV seroprevalence was very high, reaching 100%, 95% and 90% in Jura, Lucerne and Thurgau, respectively. However, as SBV is not notifiable in Switzerland, no data was available on the extent of SBV circulation in previous years. Previous studies have demonstrated that SBV serological assays can detect antibodies induced by related Simbu serogroup viruses, highlighting the limitations of using SBV ELISA results alone to specifically detect SBV circulation or incursions of new but related viruses [27].

The absence of detectable SBV RNA in animals with high levels of SBV antibodies might be time-dependent, as viraemia is relatively short, making direct virus genome detection difficult once the acute phase has passed. Consequently, RT-qPCR-negative results cannot exclude recent SBV infection. Nevertheless, the molecular results were particularly informative. Of the 16 animals initially selected because of clinical disease or weak/negative SBV ELISA results, six were positive by both pan-Simbu RT-qPCR and the SBV L-segment RT-qPCR but negative in RT-qPCRs targeting the SBV M and S segments. This pattern is unlikely to represent a SBV infection in which all three segments would be expected to be RT-qPCR positive. Instead, it suggested that thepan-Simbuu assay and L-segment assay detected a genetically related *Orthobunyavirus*, while the absence of detection of the SBV M and S segments indicated substantial genomic divergence from SBV in the genomic regions targeted by the primers for these segments.

It is known that isolation and propagation of SBV from viremic serum in cell culture often results in low virus titer during the initial passage [28]. Several additional passages may be required before rapid CPE appears and virus titer increases to sufficient levels. Based on this, we decided to compare virus isolations in parallel using BHK-21 cells, which are commonly used for SBV isolation and SK-6 cells, which have previously been shown to be more efficient for SBV replication and to yield higher virus titers after fewer passages [29]. Interestingly, in our study the isolation of SHAV was more efficient in BHK-21 cells than SK6 cells, showing that the cellular tropism and replication of SHAV may differ from those described for SBV.

The genetic distance provides strong evidence that the novel *Orthobunyavirus* detected during the 2026 outbreak is genetically more closely related to SHAV than SBV. The segment-wise phylogenetic analyses further support the closer relationship of the 2026 outbreak virus to SHAV. Nevertheless, the phylogenetic topology differed among the three segments, indicating that the evolutionary development of the novel virus may not be identical across the genome. Most notably, the S segment formed a distinct subclade within the broader SHAV lineage. This clustering demonstrates that the novel virus is genetically distinct from previously characterized SHAV sequences, while still remaining in the SHAV lineage. Phylogenetic analyses showed a close evolutionary relationship between SBV and SHAV, although their evolutionary history and the mechanisms underlying their diversification remain incompletely understood. Furthermore, reassortment is an important mechanism of evolution among *Orthobunyaviruses*; therefore, characterization of all three genome segments is essential for determining the genetic relationships and potential reassortment history of newly detected viruses.

Following detection of the initial index case by PCR, the virus has rapidly expanded its geographic distribution, with confirmed infections now reported in 110 farms distributed across 21 Swiss cantons (25 August 2026), including Ticino, south of the Alps. The detection of positive cases on both sides of the Alpine barrier indicates that the virus has successfully crossed this major geographic barrier. Given that such barriers are meaningful for dispersal of insect vectors, it is unlikely that vector movement alone accounts for the wide geographical spread of the virus. Instead, animal movement, particularly of infected animals, may have played an important role in facilitating virus introduction and spread across the Alpine region. The broad geographic distribution within a relatively short period is consistent with efficient transmission of an arbovirus within a susceptible ruminant population. The emergence of SBV in Europe in 2011 was associated with acute febrile disease and reduced milk production in adult cattle and with abortions, stillbirths and congenital malformations in ruminants [30]. The current Swiss findings demonstrate that the emergence of another closely related Simbu-group *Orthobunyavirus* can produce similarly extensive geographic distribution and clinical signs.

Members of the Simbu serogroup are mainly associated with transmission by *Culicoides* biting midges; however, mosquitoes have also been implicated in the epidemiology of some viruses within this group. Experimental studies of, for example, SHUV or SBV have shown significant differences in vector competence between biting midges and mosquitoes [31,32,33]. SHUV RNA has been detected in field-collected mosquitoes, representing several genera, including *Mansonia*, *Culex*, *Aedes* and *Anopheles* in South Africa between 2014 and 2018 [34], while SBV RNA has been mainly detected in *Culicoides obsoletus* [35,36,37]. Detection of viral RNA in field-collected mosquitoes does not by itself confirm vector competence, virus replication within vectors, or successful transmission. Therefore, to determine the vector species enabling SHAV spread in Europe, vector competence studies, including both biting midges and mosquitoes, will be of critical importance.

Detection of viral RNA in brains of aborted bovine fetuses is of particular significance and provides initial evidence of transplacental transmission and fetal infection; however, further assays like immunohistochemistry or in situ hybridization demonstrating viral antigens or RNA in affected tissue would be necessary to support the hypothesis that viral replication contributed to the abortion. Nevertheless, fetal infection is a characteristic feature of several Simbu serogroup *Orthobunyaviruses*. SHAV itself has previously been associated with congenital malformations in cattle, while SBV is well known to cause abortions, stillbirth and congenital malformations following transplacental infection [38].

Finally, the detection of the SHAV raises important questions regarding the epidemiology, host range and evolutionary history of this virus. The extensive geographic distribution within Switzerland, together with reports of positive cases in Germany, France, Netherlands and Austria, suggests that transmission was already well established beyond the initially recognized affected farms. For arthropod-borne viruses, further investigation of Culicoides midges and Aedes/Culex mosquitoes, including their infection status, seasonal abundance, vector competence, and geographic distribution, will be essential to elucidate the mechanisms underlying virus emergence and spread. Furthermore, demonstrated capacity for genome reassortment among the Simbu serogroup and continued co-circulation of newly identified SHAV and SBV in Europe can create possible genetic diversifications and cause the emergence of novel reassorted viruses. Although direct evidence for reassortment between SBV and SHAV has not been described yet, the close evolutionary relationship between both underscores the importance for continued genomic surveillance to monitor viral evolution and identify potential reassortment.

## Figures and Tables

**Table 1:** Comparison of SBV ELISA and serum neutralization test results. The table shows the comparison between SBV-specific antibody detection by ELISA and serum neutralization testing in 10 selected serum samples. ELISA results are expressed as the sample-to-positive (S/P) percentage, while SNT results are reported as the serum dilution corresponding to the neutralization endpoint, calculated using the Spearman–Kärber method.

Probe ID	ELISA SBV S/P %	SNT Titer
1. 261,239.20	207	>1:256
2. 261,239.43 *	10	<1:4
3. 261,239.44 *	124	<1:4
4. 261,232.1	213	1:108
5. 261,219.1	213	1:64
6. 261,216.16	188	1:38
7. 261,208.1	162	1:4
8. 261,197.2	222	1:108
9. 261,197.4	205	1:215
10. 261,197.9	213	1:16

* Samples from young calves.

**Table 2:** Overview of sequenced samples, including GenBank accession numbers, sample material, total raw reads generated, reads mapped to SHAV, and mean sequencing depth.

Sample	Accession Number Seg L/M/S	Material	Reads Sequenced	Reads Mapped	Mean Depth
LU_261239_1	PZ831155/PZ831159/PZ831163	Serum	217′238′193	24′666′501	270′616
LU_261239_3	PZ831156/PZ831160/PZ831164	Serum	325′054′039	146′189′349	741′480
TG_261216_18	PZ831157/PZ831161/PZ831165	Serum	179′790′516	52′787′023	487′052
TG_261216_36	PZ831158/PZ831162/PZ831166	EDTA-Blood	158′713′875	33′490	358

## Data Availability

The nucleotide sequences of SHAVs from this study have been registered at GenBank under the following accession numbers: PZ831155-PZ831166. Raw sequencing reads are available under project number PRJEB124419 at ENA.
